# Patterns and predictors of violence against children in Uganda: a latent class analysis

**DOI:** 10.1136/bmjopen-2015-010443

**Published:** 2016-05-24

**Authors:** Kelly Clarke, Praveetha Patalay, Elizabeth Allen, Louise Knight, Dipak Naker, Karen Devries

**Affiliations:** 1 London School of Hygiene and Tropical Medicine, London, UK; 2 University College London, London, UK; 3 Raising Voices, Kampala, Uganda

**Keywords:** Childhood violence, Child mental health, Emotional violence, Sexual violence, Physical violence, Uganda

## Abstract

**Objective:**

To explore patterns of physical, emotional and sexual violence against Ugandan children.

**Design:**

Latent class and multinomial logistic regression analysis of cross-sectional data.

**Setting:**

Luwero District, Uganda.

**Participants:**

In all, 3706 primary 5, 6 and 7 students attending 42 primary schools.

**Main outcome and measure:**

To measure violence, we used the International Society for the Prevention of Child Abuse and Neglect Child Abuse Screening Tool—Child Institutional. We used the Strengths and Difficulties Questionnaire to assess mental health and administered reading, spelling and maths tests.

**Results:**

We identified three violence classes. Class 1 (N=696 18.8%) was characterised by emotional and physical violence by parents and relatives, and sexual and emotional abuse by boyfriends, girlfriends and unrelated adults outside school. Class 2 (N=975 26.3%) was characterised by physical, emotional and sexual violence by peers (male and female students). Children in Classes 1 and 2 also had a high probability of exposure to emotional and physical violence by school staff. Class 3 (N=2035 54.9%) was characterised by physical violence by school staff and a lower probability of all other forms of violence compared to Classes 1 and 2. Children in Classes 1 and 2 were more likely to have worked for money (Class 1 Relative Risk Ratio 1.97, 95% CI 1.54 to 2.51; Class 2 1.55, 1.29 to 1.86), been absent from school in the previous week (Class 1 1.31, 1.02 to 1.67; Class 2 1.34, 1.10 to 1.63) and to have more mental health difficulties (Class 1 1.09, 1.07 to 1.11; Class 2 1.11, 1.09 to 1.13) compared to children in Class 3. Female sex (3.44, 2.48 to 4.78) and number of children sharing a sleeping area predicted being in Class 1.

**Conclusions:**

Childhood violence in Uganda forms distinct patterns, clustered by perpetrator and setting. Research is needed to understand experiences of victimised children, and to develop mental health interventions for those with severe violence exposures.

**Trial registration number:**

NCT01678846; Results.

**Strengths and limitations of this study**

- This is one of the first studies to explore the basic patterning of exposure to different forms of violence, from different perpetrators, in a low-income setting with a high rate of childhood violence.
- This study uses survey data from a large representative sample of primary school children in Uganda, however, results should not be generalised to those outside this group.
- Some children may not have felt comfortable disclosing exposure to sexual violence, possibly due to fear or embarrassment associated with these experiences.
- Owing to the cross-sectional nature of the survey data, we were unable to establish causal relationships between violence exposures and predictors, including mental health.

## Introduction

Violence against children, including physical, sexual and emotional abuse, is a global concern with important health consequences including depression, suicidal behaviour, sexually transmitted infections, risky sexual behaviour and death.^1–5^ Child victims of violence have worse educational outcomes compared to their non-abused peers, and are at increased risk of financial and employment problems in later life.^5^ Children with disabilities and children from socioeconomically disadvantaged families are at increased risk of violence compared to their peers.^6^ ^7^ In many settings, rates of sexual violence are higher in girls, while boys may experience more physical violence.^7–12^

Evidence suggests that some children who are exposed to violence are polyvictimised, meaning they are exposed to more than one form of abuse.^13^ Such victims are more likely to report social and economic problems, post-traumatic stress, physical health problems and suicide behaviours, compared to those exposed to only one form of violence.^5^ ^14^ Exposure to violence in multiple contexts, the nature of the relationship with the perpetrator and the frequency and severity of violence are also likely to affect children's outcomes.^13^ ^15–18^

To date, few studies have explored the basic patterning of exposure to different forms of violence, from different perpetrators, particularly in low and middle-income settings. Two studies, conducted in Denmark and the USA, showed that childhood violence exposures could be grouped according to the type of violence (ie, physical, sexual or emotional), with an additional group of polyvictimised children.^19^ ^20^ It also remains unclear how different patterns of exposure relate to health and educational outcomes. This is important, as recent global data suggest substantial variation in the prevalence of exposure to different forms of violence, and variation in patterns of perpetration.^21^ In high-income countries, the prevalence of emotional violence is around 10%, while physical violence estimates range from 4% to 16% per year.^7^ Rates are higher in low-income countries, especially in Africa, where 83%, 64% and 43% of children experience emotional, and moderate and severe physical abuse by their parents, respectively,^21^ and lifetime exposure to sexual violence is 23% (95% CI CI 9% to 33%).^2^

Our paper focuses on violence against children in Uganda. A recent study conducted in one Ugandan district found that over 90% of children have experienced physical violence in their lifetime, over half report emotional abuse and 4% of boys and 13% of girls report sexual abuse.^22^ Ninety-three per cent of boys and 94% of girls have ever experienced physical abuse by school staff.^22^ Using data from this study, we aim to identify and characterise patterns of physical, emotional and sexual violence against children in this setting. As far as we are aware, no previous literature has examined these patterns among children in Uganda.

## Methods

### Setting

We used baseline survey data from the Good Schools Study.^23^ ^24^ This study was a cluster-randomised controlled trial of the Good School Toolkit, an intervention developed by Raising Voices, a Ugandan non-profit organisation.^24^ The intervention used a whole school approach and was designed to reduce violence from staff to students and also between students. The primary outcome was physical violence against children by school staff.^23^ The baseline survey took place in June and July 2012, in Luwero District, in the Central Region of Uganda. Luwero comprises both rural and urban areas, and has a population of 458 158.^25^ The local language is Luganda. In the 1980s, Luwero district was the site of an insurgency, which involved large-scale murder and starvation of civilians.^26^

### Sampling

Our sampling strategy is described elsewhere.^23^ Briefly, we sampled children through primary schools. From the 268 primary schools in the district, we excluded 97 small schools (with <40 registered primary 5 students) and 20 schools with existing governance interventions. The remaining 151 schools were attended by 80% of all primary 5, 6 and 7 students in Luwero. These schools were stratified based on the gender ratio of students as follows: 13 schools with >60% girls; 14 schools with >60% boys; and 124 schools with approximately equal numbers of girls and boys. We randomly selected 42 schools, proportional to the size of the stratum. From each school we randomly sampled 130 students from primary 5, 6 and 7, and invited them to participate in the survey. In schools with <130 students, all students were invited to participate. Research teams spent 3–6 days in each school to conduct the survey and made at least one repeat visit to find students who had been absent during that time. We were able to collect data from 77% of sampled students; 19% of students were absent from school.

### Ethics

Consent and child protection procedures for this study are described in detail elsewhere.^23^ ^27^ Head teachers at each participating school informed staff, students and parents about the study. We notified parents of children at participating schools about the study in several different ways.^23^ Children were allowed to provide consent, rather than assent, because: (1) it was possible to obtain informed consent using a consent form containing a simple description of the study procedure; (2) parents were given the opportunity to opt their children out of the study; and (3) this consent procedure was approved by two independent ethics committees. If children were unable to provide informed consent (eg, they had a disability that meant they could not read the consent form or hear it read aloud, or they did not understand the study procedures described), they were automatically excluded from the study. We developed and implemented a child protection plan to support and link vulnerable children with appropriate services. A trained counsellor was available to any child requesting counselling.

### Data collection and instruments

Through in person interviews, we collected data on child sociodemographics, absence from school, educational performance, mental health and experiences of physical, emotional and sexual violence. All items in the interview were translated into Luganda, and we conducted a three-phase review process. In the first phase, teachers, Raising Voices staff and school staff reviewed the items; in phase 2, we tested items on a sample of ∼40 children from primary schools in Kampala to check understanding and meaning; finally, we surveyed 697 children from Kampala primary schools to assess item distribution and pilot study procedures.

We assessed academic performance through educational tests including word recognition (scoring 1–40), timed reading (1–62) and reading comprehension (1–5). We also administered the following group-based tests: silly sentences (testing reading and cognitive ability, scoring 1–20), spelling (1–20) and maths (1–40). A global academic performance score was calculated by dividing the distribution of scores for each test into thirds and allocating one point to children scoring in the bottom third, two points to those scoring in the middle third and three points to those in the top third. We then calculated the mean of these scores over the number of tests completed for each child and labelled the 10% of children with the lowest mean scores as ‘low educational performers’.

To assess children's mental heath, we used the Strengths and Difficulties Questionnaire (SDQ), which has been extensively translated and used in diverse settings.^28^ The questionnaire comprises five subscales: emotional symptoms, conduct problems, hyperactivity/inattention, peer relationship problems and prosocial behaviour. Each subscale comprises five statements, and children were asked whether they felt the statement was not true (0), somewhat true (1) or certainly true (2). We calculated a SDQ total difficulties score by summing individual subscale scores (excluding the prosocial subscale).

To screen for physical, sexual and emotional violence, we used the International Society for the Prevention of Child Abuse and Neglect Child Abuse Screening Tool-Child Institutional with additional items from the WHO Multi Country Study on Women's Health and Domestic Violence against Women.^29^ ^30^

### Analysis

We used latent class analysis with maximum likelihood estimation to identify distinct patterns or latent classes of violence experienced by children in the sample.^31^ Based on violence items included in the interview, we constructed 14 variables for inclusion in the latent class model (table 1). Physical violence variables incorporated information about perpetrator and severity of violence. For emotional violence variables, we used frequency as a proxy for severity since it was unclear which emotional violence acts children perceived as most severe. Sexual violence variables were coded as binary, due to low rates of reported sexual violence in the sample.

**Table 1:** Coding of violence variables

Type of violence	Variables	Items	Coding
Emotional violence	Emotional abuse by a parent; emotional abuse by a peer; emotional abuse by school staff; emotional abuse by a non-parent relative; emotional abuse by others*	Cursed, insulted, shouted at or humiliated you? Referred to your skin colour, gender, religion, tribe or health problems you have in a hurtful way? Stopped you from being with other children to make you feel bad or lonely? Tried to embarrass you because you were an orphan or without a parent? Embarrassed you because you were unable to buy things? Stole or broke or ruined your belongings? Threatened you with bad marks that you didn't deserve? Accused you of witchcraft? Made you stay outside, for example, in the heat or rain to punish you? Taken your food away from you as punishment?	Coded 2 if any item was experienced many times in the past 12 months or before the past 12 months; 1 if any item was experienced a few or once in the past 12 months or before the past 12 months; 0 if answered no to all items
Physical violence	Physical abuse by a parent; physical abuse by a peer; physical abuse by school staff; physical abuse by a non-parent relative; physical abuse by others*	*Severe physical violence:* Choked you? Severely beat you up? Tried to cut you purposefully with a sharp object? Burnt you as punishment? Moderate physical violence: hurt you or caused pain to you? Slapped you with a hand on your face or head as punishment? Slapped you with a hand on your arm or hand? Twisted your ear as punishment? Twisted your arm as punishment? Pulled your hair as punishment? Hit you by throwing an object at you? Hit you with a closed fist? Hit you with a stick? Caned you? Kicked you? Knocked you on the head as punishment? Made you dig, slash a field or do other labour as punishment? Hit your fingers or hands with an object as punishment? Crushed your fingers or hands as punishment? Made you stand or kneel in a way that hurts to punish you? Forced you to do something that was dangerous? Tied you up with a rope or belt at school?	Coded 2 if any severe physical violence item was experienced; 1 if any moderate item was experienced; 0 if answered no to all items
Sexual violence	Sexual abuse by a peer; sexual abuse by a relative; sexual abuse by school staff; sexual abuse by others	Teased you or made sexual comments about your breasts, genitals, buttocks or other body parts? Touched your body in a sexual way or in a way that made you uncomfortable? By ‘sexual way,’ we mean touching you on your genitals, breasts or buttocks. Showed you pictures, magazines, or movies of people or children doing sexual things? Made you take off your clothes when it was not for a medical reason? Opened or took their own clothes off in front of you when they should not have done so? Kiss you when you didn't want to be kissed? Make you touch their genitals, breasts or buttocks when you didn't want to? Touched your genitals, breasts or buttocks when you didn't want them to? Given you money or things in exchange for doing sexual things? Involved you in making sexual pictures or videos? Threatened or pressured you to have sex or do sexual things with them? Actually made you have sex with them by threatening or pressuring you or by making you afraid of what they might do? Made you have sex with them by physically forcing you (have sex with you)?	Coded 1 if answered yes to any item; 0 if no to all items

We determined the optimum number of classes by considering the appropriateness and usefulness of classes, in addition to examining well-established statistical model selection criteria including model fit, neatness of classification and model comparison.^31^ ^32^ We used the sample size Adjusted Bayesian Information Criterion (A-BIC), a measure of relative goodness of fit, where lower values indicate better fit. We also used the Lo-Mendell-Rubin Likelihood Ratio Test (LMR-LRT) of goodness of fit, where a non-significant p value indicates that the model with one or fewer classes is preferable, and entropy as a measure of classification quality, with higher entropy values being preferable.

In order to identify predictors of individual class membership, we conducted a multinomial logistic regression analysis, accounting for clustering at the school level. Since the entropy value was relatively low in our selected latent class model, we also conducted a sensitivity analysis including weights for class probability estimates in the regression model. We excluded 77/3706 (2.1%) individuals from the regression analysis, due to missing data. Although the percentage of excluded individuals differed significantly by class (Class 1: 3.2%; Class 2: 1.0%; Class 3: 2.2%; χ^2^ 9.50, p=0.009), exclusion of such a small proportion of the sample is unlikely to have affected the overall results.

Latent class modelling was conducted using MPlus V.7.0 software.^33^ For all other statistical analyses we used Stata V.12.^34^

## Results

### Characteristics of children

We interviewed 3706 primary school children in 42 schools in Luwero District. Their mean age was 13 years (SD 1.5), ranging from 7 to 18 years. Half (52.3%, 1937/3706) the children sampled were girls. Several indicators suggested a substantial proportion of children were from socioeconomically disadvantaged homes: half (52.9%, 1959/3706) had eaten less than three meals on the previous day and 34.8% (1287/3706) had worked for money outside school. Seven per cent (271/3706) said they had a physical or mental disability. Further information about demographic characteristics of the sample is provided elsewhere.^22^

The mean total SDQ score for the sample was 9.3 (SD, 5.3). Rates of reported violence among children were high. Almost all children (94.4%, 3500/3706) had experienced some form of physical violence, and 58.3% (2160) reported experiencing emotional violence. The rate of sexual violence was lower, at 8.9% (329/3706).

### Classifying and characterising violence exposures

We ran latent class models with two to six latent classes. A-BIC, entropy values, as well as the LMR-LRT (table 2) indicated that the three-class model was statistically the most feasible. In addition, the three-class model provided the most meaningful classifications of violence and was hence chosen for further analysis.

**Table 2:** Statistical criteria for latent class modes with 2–6 latent classes

Number of classes	A-BIC	Entropy	LMR-LRT
2	35615.057	0.514	1119.196*
3	35084.842	0.602	647.894*
4	35004.205	0.585	200.585
5	34986.703	0.655	137.768
6	34990.520	0.687	116.557

*p<0.05.

A-BIC, Adjusted Bayesian Information Criterion; LMR-LRT, Lo-Mendell-Rubin Likelihood Ratio Test.

Values in table 3 represent the probability, by class, that a child had experienced a given subcategory of violence. The largest class was Class 3, comprising 54.9% (2035/3706) of the children in the sample. The probability of experiencing physical violence by school staff in this class was high (moderate physical violence 87.4%; severe physical violence 0.6%), while probabilities associated with other forms of violence were relatively low.

**Table 3:** Probability estimates of violence exposure by class

	Class 1	Class 2	Class 3
Number (% total sample)	696 (18.8)	975 (26.3)	2035 (54.9)
*Emotional violence*			
Abused by parent			
Severe	10.0	0.7	0.0
Moderate	19.0	3.1	0.1
None	71.0	96.2	99.9
Abused by peers			
Severe	9.9	30.6	2.2
Moderate	11.9	51.8	13.0
None	78.2	17.6	84.8
Abused by school staff			
Severe	13.0	14.7	1.4
Moderate	38.5	36.7	9.4
None	48.4	48.6	89.3
Abused by relatives			
Severe	8.3	0.0	0.2
Moderate	11.8	1.2	1.4
None	80.0	98.8	98.3
Abused by others			
Severe	8.8	0.6	0.9
Moderate	17.7	0.9	3.7
None	73.6	98.5	95.4
*Physical violence*			
Abused by parent			
Severe	10.0	0.2	0.0
Moderate	46.4	11.5	13.3
None	52.6	88.3	86.7
Abused by peers			
Severe	0.0	1.1	0.1
Moderate	24.7	64.5	12.6
None	75.3	34.3	87.3
Abused by school staff			
Severe	16.4	13.0	0.6
Moderate	83.6	86.8	87.4
None	0.0	0.2	12.0
Abused by relatives			
Severe	0.7	0.0	0.0
Moderate	11.5	1.6	1.1
None	87.8	98.4	98.9
Abused by others			
Severe or moderate	5.7	1.9	2.3
None	94.3	98.1	97.7
*Sexual violence*			
Abused by peers			
Severe or moderate	2.7	9.0	0.5
None	97.3	91.0	99.5
Abused by relatives (including parents)			
Severe or moderate	1.4	0.3	0.1
None	98.6	99.7	99.9
Abused by others			
Severe or moderate	13.8	3.4	0.4
None	86.2	96.6	99.6
Abused by school staff			
Severe or moderate	4.8	3.8	0.2
None	95.2	96.2	99.8

Children in Classes 1 and 2 were polyvictimised, and experienced more severe forms of violence. In addition to a high probability of experiencing any form of moderate physical violence by school staff (Class 1 83.6%; Class 2 86.8%), children in Classes 1 and 2 were more likely to have experienced severe incidents of physical violence (ie, choking, burning or being severely beaten or cut) and emotional violence at school compared to children in Class 3.

Children in Class 1 had a higher probability of having experienced severe and moderate forms of emotional and physical violence by parents and relatives (including siblings) compared to their peers. Children in this class also experienced more sexual and emotional abuse by others, namely, boyfriends, girlfriends and unrelated adults outside school.

Children in Class 2 had a higher probability of experiencing severe and moderate forms of physical, emotional and sexual violence by peers (male and female school students) compared to children in Classes 1 and 3.

Table 4 shows further sociodemographic characteristics of the three classes of violence exposures. Compared to children in Classes 2 and 3, Class 1 comprised a higher proportion of girls and school boarders, and all children in this class had experienced some form of physical violence. Ninety-four per cent (653/696) and 24.4% (170/696) of children in Class 1 had experienced emotional and sexual violence, respectively, compared to 26.7% (544/2035) and 0.8% (17/2035) of children in Class 3.

**Table 4:** Characteristics of children with violence exposures

	Class 1	Class 2	Class 3	Overall sample
Number (% total sample)	696 (18.8)	975 (26.3)	2035 (54.9)	3706
Gender female (%)	494 (71.0)	439 (45.0)	1004 (49.3)	1937 (52.3)
Age*				
Mean (SD)	13.0 (1.5)	13.0 (1.5)	13.0 (1.4)	13.0 (1.5)
Median (range)	13 (9–18)	13 (9–18)	13 (7–18)	13 (7–18)
Number of meals eaten yesterday†				
1 meal	116 (16.7)	157 (16.1)	243 (12.0)	516 (13.9)
2 meals	268 (38.5)	393 (40.3)	782 (38.5)	1443 (39.0)
3+ meals	312 (44.8)	425 (43.6)	1009 (49.6)	1746 (47.1)
Number of children sharing sleeping area				
0	104 (14.9)	126 (12.9)	248 (12.2)	478 (12.9)
1	234 (33.6)	291 (29.9)	532 (26.1)	1057 (28.5)
2–4	270 (38.8)	422 (43.3)	918 (45.1)	1610 (43.4)
5–7	27 (3.9)	47 (4.8)	82 (4.0)	156 (4.2)
8+	61 (8.8)	89 (9.1)	255 (12.5)	405 (10.9)
Ever worked for money‡	240 (34.5)	408 (41.9)	639 (31.4)	1287 (34.8)
Transport to school§				
Other	25 (3.6)	21 (2.2)	93 (4.7)	139 (3.8)
Walking alone	169 (24.5)	240 (25.4)	481 (24.2)	890 (24.6)
Walking with someone you know	423 (61.4)	622 (65.9)	1269 (63.8)	2314 (63.9)
Boarded at school	72 (10.5)	61 (6.5)	146 (7.3)	279 (7.7)
Disability	55 (7.9)	87 (8.9)	129 (6.3)	271 (7.3)
Absent on 1 or more days in past week¶	159 (23.6)	240 (24.8)	373 (18.7)	772 (21.2)
Low performer on educational tests	63 (9.1)	103 (10.6)	212 (10.4)	378 (10.2)
SDQ total score M (SD)	10.6 (5.4)	10.9 (5.5)	8.2 (4.9)	9.3 (5.3)
Physical violence**	696 (100)	975 (100)	1829 (89.9)	3500 (94.4)
Emotional violence**	653 (93.8)	963 (98.8)	544 (26.7)	2160 (58.3)
Sexual violence**	170 (24.4)	142 (14.6)	17 (0.8)	329 (8.9)

*N=3701.

†N=3705.

‡N=3704.

§N=3622.

¶N=3635.

**Refers to violence by any perpetrator.

SDQ, Strengths and Difficulties Questionnaire.

Compared to their peers, a smaller proportion of children in Class 2 ate three meals on the previous day, and a higher proportion had ever worked for money, suggesting they may be more socioeconomically disadvantaged compared to children in other classes. Children in Class 2 reported the highest rate of absences on one or more days during the week prior to the survey.

SDQ scores were higher in children in Classes 1 and 2, suggesting that these children experienced more mental health symptoms compared to children in Class 3.

### Predicting violence class membership

We conducted a multinomial logistic regression analysis to identify predictors associated with violence class membership, using Class 3 as the reference class (table 5). We found that girls were almost three and a half times more likely to be in Class 1 than in Class 3 (relative risk ratio RRR 3.44, 95% CI 2.48 to 4.78, p<0.001). Conversely, children who shared their sleeping area with increasing numbers of children were less likely to be in Class 1. The relationship persisted when data from school boarders (N=279) were omitted from the analysis. In contrast, lower age was significantly associated with Class 2 membership (RRR 0.94, 95% CI 0.88 to 1.00, p=0.044).

**Table 5:** Predictors of violence exposure

	Class 3	Class 1			Class 2		
		RRR (SE)	95% CI	p Value	RRR (SE)	95% CI	p Value
Sex (female)	Reference group	3.44 (0.58)	2.48 to 4.78	<0.001	0.95 (0.11)	0.75 to 1.20	0.663
Age		1.01 (0.04)	0.93 to 1.09	0.819	0.94 (0.03)	0.88 to 1.00	0.044
Ever worked for money (yes)		1.97 (0.24)	1.54 to 2.51	<0.001	1.55 (0.15)	1.29 to 1.86	<0.001
Number of meals eaten yesterday							0.261*
3 meals		Reference category					
2 meals		1.09 (0.13)	0.88 to 1.37	0.427	1.02 (0.10)	0.85 to 1.24	0.774
1 meal		1.30 (0.20)	0.96 to 1.77	0.089	1.21 (0.17)	0.91 to 1.60	0.187
Number of children sharing sleeping area							0.001*
0		Reference category					
1		1.00 (0.12)	0.79 to 1.26	0.987	1.11 (0.12)	0.90 to 1.38	0.330
2–4		0.62 (0.09)	0.47 to 0.81	0.001	0.89 (0.10)	0.71 to 1.12	0.329
5–7		0.65 (0.16)	0.39 to 1.06	0.086	1.04 (0.24)	0.66 to 1.65	0.868
8+		0.53 (0.13)	0.33 to 0.85	0.009	0.70 (0.16)	0.45 to 1.08	0.109
Disability (yes)		1.04 (0.17)	0.75 to 1.43	0.818	1.17 (0.15)	0.90 to 1.51	0.235
Absent on 1 or more days in past week		1.31 (0.17)	1.02 to 1.67	0.031	1.34 (0.14)	1.10 to 1.63	0.004
Low performer on educational tests (yes)		0.70 (0.13)	0.49 to 1.02	0.063	0.78 (0.13)	0.56 to 1.09	0.146
SDQ total score		1.09 (0.01)	1.07 to 1.11	<0.001	1.11 (0.01)	1.09 to 1.13	<0.001

*Overall p value for the categorical variable.

SDQ, Strengths and Difficulties Questionnaire.

Several predictors were associated with both Class 1 and Class 2 violence exposures, including having ever worked for money (Class 1 RRR 1.97, 95% CI 1.54 to 2.51, p<0.001; Class 2 RRR 1.55, 95% CI 1.29 to 1.86, p<0.001) and absenteeism in the previous week (Class 1 RRR 1.31, 95% CI 1.02 to 1.67 p=0.031; Class 2 RRR 1.34 95% CI 1.10 to 1.63 p=0.004). SDQ score was higher in children in Class 1 (RRR 1.09, 95% CI 1.07 to 11, p<0.001) and Class 2 (RRR 1.11 95%CI 1.09 to 1.13, p RRR 1.09, 95% CI 1.07 to 11, p<0.001), however, disability and educational performance did not predict violence class membership. Weighting the regression analysis by class probabilities did not change the results.

## Discussion

### Summary of main findings

School children in Uganda are at high risk of all forms of violence, particularly physical and emotional violence by school staff, peers and parents. We identified three classes of children with distinct violence exposures defined by perpetrator and setting. All classes experienced high rates of violence by school staff despite a ban on corporal punishment in Ugandan schools. Class 3 was largest and characterised by physical violence perpetrated by school staff. Children in Class 1 were mainly girls who had experienced multiple and severe forms of violence at home, and sexual violence from ‘other’ perpetrators. Class 2 comprised children who had experienced a substantial amount of violence from their peers and who were likely to be slightly younger. Children in Classes 1 and 2 experienced violence in multiple contexts, for example, children in Class 1 were at risk of exposure to violence at home and in school. Children in Classes 1 and 2 also had worse mental health outcomes relative to children in Class 3.

### Comparison with other studies

Few studies have examined childhood violence using latent class analysis. A retrospective study of young adults in Denmark identified four violence classes: non-abused, psychologically abused, sexually abused and a class experiencing multiple abuse types.^19^ Nooner *et al* characterised four exposures to physical and sexual violence among children in the USA, however, emotional violence was not included.^20^ In both these studies, the majority of sampled children had not been exposed to violence, and information on perpetrator was not incorporated into the analyses. In contrast, violence classes in our study were mainly defined by perpetrator and setting, rather than type or severity of violence. Since the prevalence of violence in our sample was comparatively high, we did not identify a non-abused class and polyvictimisation was common.

We found that children in Classes 1 and 2 had more mental health difficulties than children in Class 3. This is consistent with studies suggesting that individuals with cross-contextual violence exposures by multiple perpetrators may have poorer mental health.^17^ ^18^ Witnessing violence —for example, community or domestic violence—may reduce or strengthen the association between children's violence exposures and mental health, however, we did not investigate these effects in the study.^35^ ^36^ A school-based intervention in Luwero District reduced violence against children by school staff but did not significantly improve children's mental health.^24^ One possible explanation for this is that, in addition to violence by school staff, many children were also experiencing violence in other contexts, which was not specifically addressed by the intervention. This suggests there is a need for treatment of the effects of multiple co-occurring types of violence from multiple perpetrators in this setting, which is directly related to the patterns of violence described in this study.

Our finding that girls are more likely to experience multiple and severe forms of violence from family members, and sexual violence from others, is consistent with national studies reporting higher rates of domestic violence against adult women compared to those against men in many African settings.^37^ Girls may be at greater risk due to spending more time in the home and being physically weaker than their brothers. Son preference may also be a factor in certain cultural contexts.^38–41^ Early intervention is necessary to prevent re-victimisation of and long-term effects for girls, including interpersonal violence, sexual coercion, alcohol and drug abuse and mental health problems.^42^ ^43^

Children in Class 2, which was characterised by a higher risk of peer-related violence, were more likely to have been absent in the previous week, suggesting absenteeism may be a coping mechanism. These children were at higher risk of sexual violence compared to their peers, and had poorer mental health than children with school staff physical violence exposures in Class 3. Long-term effects of childhood sexual violence include substance misuse, early sexual debut, more sexual partners, trading sex for financial gain and less use of contraception, as well as interpersonal difficulties, post-traumatic stress disorder and suicidal behaviour.^44–51^ Our results imply that interventions to reduce peer-related violence among primary school children could be beneficial for mental health, as well as for sexual and reproductive health in this population.

Although previous studies have reported associations between educational performance and violence exposure, low performance on educational tests did not predict class membership in our study.^10^ ^52^ This could be because children respond differently to violence, for example, some children exposed to violence at home may immerse themselves in their schoolwork whereas others may withdraw. Furthermore, children may be victimised by peers because they underachieve at school whereas others may be bullied for overachieving. Additional research is needed to investigate relationships between educational performance and violence exposure among children.

### Implications

These novel findings suggest that patterning of childhood violence in this context is clustered by perpetrator and setting. Research is needed to understand pathways to perpetration, and how the nature of the perpetrator's relationship to the victim might determine the health and social effects of violence. Interventions targeting groups of perpetrators, rather than victims, may be beneficial. Since most of the children in our study were exposed to school staff physical and/or peer-related violence, school-based interventions for teachers and students could potentially reduce the main sources of childhood violence in this setting. Interventions targeting perpetration by parents could potentially reduce some important violence exposures for children in Class 1.^53^

Further research is necessary to understand associations between different forms of violence for the group of mainly girls experiencing severe violence at home and sexual abuse from other perpetrators. For example, are girls from violent families more vulnerable to perpetrators of sexual violence outside the home? Alternatively, are girls who have been sexually victimised outside the home more likely to be punished and stigmatised by their family? Understanding these pathways would help to design effective interventions to support children in this class.

### Limitations

Our study has several limitations. First, it is likely that sexual violence was under-reported. Subsequent surveys using alternative approaches to asking about sexual violence have estimated higher rates in the same population of children.^24^ Second, the latent classes of violence exposure identified in this study are statistical constructs, requiring further validation and characterisation using qualitative research methods. Third, our study includes only children enrolled in primary school who attended school during the survey. Results might not be generalisable to children who were absent or not enrolled, and who may represent a more vulnerable group. Last, due to the cross-sectional nature of our data, we were unable to establish causal relationships between class membership and predictors.

## Conclusions

Overlapping experiences of physical, emotional and sexual violence, clustered by perpetrator and setting, are common among children in Uganda. Future interventions addressing both, perpetrators and victims are necessary for safer schools and homes, and to improve children's mental health.
